# Environmental Exposure to Trace Metals and Fluoride in Drinking Water and Soil: A Study Protocol Evaluating Multi-Index Dental Pathologies in Mining-Affected Towns of Mpumalanga, South Africa

**DOI:** 10.3390/mps9050151

**Published:** 2026-10-19

**Authors:** Mnqweno Funcuza, Bheki T. Magunga, Phoka C. Rathebe, Thokozani P. Mbonane

**Affiliations:** Department of Environmental Health, Faculty of Health Sciences, Doornfontein Campus, University of Johannesburg, Johannesburg 2006, South Africa; bhekim@uj.ac.za (B.T.M.); prathebe@uj.ac.za (P.C.R.)

**Keywords:** trace metals, environmental exposure, dental fluorosis, enamel hypoplasia, acid mine drainage, mining communities, water quality, health risk assessment, Mpumalanga

## Abstract

Intensive mining in Mpumalanga, South Africa, risks contaminating drinking water and topsoil with toxic trace metals. While systemic toxicological impacts are well-documented, the specific relationship between this multi-matrix environmental exposure and localized dental hard tissue pathologies remains critically under-researched. This study protocol outlines a standardized, multidisciplinary approach to evaluate these oral health impacts. Field sampling and clinical screenings are scheduled across an 11-month execution timeline (January 2027 to November 2027). This cross-sectional protocol features three integrated phases: (1) Environmental Geochemistry: Drinking water (*n* = 120) from source, municipal, and point-of-use taps, alongside concurrent residential topsoil samples (*n* = 60), will be analysed for trace metals and metalloids (Pb, Cd, Cr, Mn, As, Fe, Cu, and Zn) using ICP-MS/ICP-OES, and fluoride (*F*^−^) using an Ion-Selective Electrode. (2) Clinical Assessment: Registered dental professionals will screen a resident cohort (*N* = 388) using Dean’s Index for fluorosis, the DMFT index for dental caries, and the Developmental Defects of Enamel (DDE) index. (3) Health Risk Assessment: Human health risks will be quantified via Chronic Daily Intake (*CDI*), Target Hazard Quotient (*THQ*), and cumulative Hazard Index (*HI*) models. This protocol will generate high-resolution spatial contamination maps using Inverse Distance Weighting (IDW) interpolation and establish predictive multivariate regression models correlating environmental metal burdens with clinical oral pathologies. Ultimately, it provides a legally defensible scientific baseline for provincial water quality policy adjustments and targeted public health interventions in mining-affected communities.

## 1. Introduction

The contamination of freshwater resources by trace metals is a global environmental challenge, particularly in regions where industrialization and extractive industries intersect with residential areas. In South Africa, the Mpumalanga province serves as the primary hub for coal mining and power generation, activities known to release various heavy metals and metalloids into the surrounding environment [1]. Trace metals such as lead (Pb), cadmium (Cd), arsenic (As), and manganese (Mn) can enter the human body through the ingestion of contaminated drinking water, leading to both acute and chronic health complications [2]. The exposure pathways for these contaminants are multifaceted and influenced by both environmental and infrastructural factors. Recent systematic reviews have highlighted significant source variability; while municipal tap water generally remains within standard safety limits, groundwater and surface water sources in mining regions frequently exceed permissible limits for toxic elements due to industrial discharge and urban runoff [2,3]. Furthermore, even when source water undergoes treatment, secondary pollution within aging distribution networks remains a critical concern, as structural degradation contributes to metal leaching from pipes directly to the consumer’s tap [4].

Mining-affected regions face these unique water quality challenges as Acid Mine Drainage (AMD) and industrial runoff facilitate the leaching of metals from geological formations into local water tables and surface soils [5]. The Mpumalanga Highveld region serves as the primary hub for South Africa’s coal mining and heavy industrial activities, generating substantial acid mine drainage (AMD) and diffuse runoff. Previous environmental and geological monitoring across eMalahleni, Middelburg, and Barberton has documented significant surface and groundwater contamination, with localized trace element and halogen concentrations frequently exceeding national (SANS 241) and international (WHO) drinking water thresholds [6]. Specifically, historical surveys in these catchments have reported elevated levels of fluoride (F^−^, ranging from 0.8 mg/L to >3.5 mg/L), lead (Pb, up to 0.05 mg/L), cadmium (Cd, up to 0.015 mg/L), arsenic (As, up to 0.03 mg/L), iron (Fe, exceeding 10 mg/L in AMD-impacted streams), and manganese (Mn, exceeding 2.5 mg/L) [7]. These baseline data confirm severe environmental degradation and provide the geochemical rationale for investigating multi-matrix exposure pathways in these residential populations.

The physiological impact manifests through diverse pathways; while historical research has focused on systemic effects like nephrotoxicity and neurotoxicity, the specific implications for oral health are gaining critical attention [8]. Elevated fluoride (F^−^) levels, often co-occurring with trace metals in groundwater, are the primary cause of dental fluorosis [9]. Emerging evidence also suggests that heavy metals like lead and cadmium can interfere with amelogenesis, potentially exacerbating enamel hypoplasia or altering the prevalence of dental caries [2,8].

Despite the high density of mining operations in Mpumalanga, there is a paucity of structured data correlating multi-metal exposure through drinking water and soil with clinical dental health outcomes. This protocol outlines a standardized, multidisciplinary methodology to fill this gap, integrating analytical chemistry with clinical screenings and Health Risk Assessment (HRA) frameworks [10,11]. The specific objectives of this research are:

- To determine and map the spatial distribution of fluoride and trace metal concentrations (Pb, Cd, Cr, Mn, As, Fe, Cu, Zn) in drinking water and soil across Mpumalanga towns.
- To evaluate the geochemical and anthropogenic factors contributing to the mobilization of high fluoride and trace metals into drinking water.
- To assess the prevalence of dental fluorosis and enamel hypoplasia among residents in the study areas using standardized clinical indices (Dean’s Index and DMFT).
- To analyze the correlation between environmental water quality and the clinical morbidity of dental defects, comparing high-impact mining zones to a control baseline.
- To develop an Integrated Environmental Health Surveillance Framework for the monitoring and mitigation of trace metal exposure in mining communities

## 2. Materials and Methods

### 2.1. Study Design and Ethical Considerations

This cross-sectional epidemiological and environmental health study is designed to evaluate heavy metal and fluoride concentrations in drinking water and topsoil across selected study locations and assess their direct impacts on clinical dental pathologies (dental fluorosis, developmental defects of enamel, and caries burden) among resident populations. Ethical approval for the human cohort evaluation, environmental sampling protocol, and clinical oral health examinations was granted on 22 June 2026 by the institutional Health Research Ethics Committee at the University of Johannesburg (Ref No: REC-4502-2026). Operational permissions and site access clearances are currently being sought from relevant municipal health directorates and participating public health facility/hospital CEOs across the designated study districts. In the event of administrative delays in municipal site access clearances, a staged rollout contingency plan will be executed, prioritizing initial sampling and clinical screening within academic and public health facility grounds where institutional ethical clearance provides direct operational jurisdiction.

Written informed consent (and assent for minor participants accompanied by parental/guardian consent) will be formally obtained prior to clinical enrollment and data collection. Participant recruitment, clinical oral health screenings, and multi-matrix environmental field sampling are scheduled to take place from 15 January 2027 to 30 June 2027, followed by laboratory analytical processing and health risk modeling through 30 November 2027.

To mitigate exposure misclassification resulting from the cross-sectional evaluation of historical amelogenesis windows (ages 0–8 years), participant eligibility criteria will restrict primary statistical modelling to long-term residents who have continuously resided in the study area for ≥5 years. Furthermore, a structured retrospective questionnaire will capture historical domestic drinking water sources, residential history, and point-of-use treatment habits since birth or early childhood. These variables will be incorporated as stratification criteria in secondary sensitivity analyses to isolate true historical developmental exposure.

#### Phase 0: Pre-Survey Pilot Validation Protocol

To ensure the joint reliability, analytical precision, and clinical inter-rater reproducibility of the integrated protocol within the specific biogeochemical context of the Mpumalanga Highveld, a Phase 0 pilot validation phase will be executed prior to primary data collection (*n* = 15 drinking water and topsoil samples; *N* = 30 resident participants across high-impact mining and baseline zones).

The analytical chemistry validation will focus on Matrix Effect Mitigation in Acid Mine Drainage (AMD)-impacted water matrices. Specifically, Total Ionic Strength Adjustment Buffer IV (TISAB IV) de-complexation efficiency will be benchmarked against elevated ferric (Fe^3+^) and aluminum (Al^3+^) matrix interferences to ensure accurate fluoride (F^−^) recovery rates (95–105%) using the Ion-Selective Electrode (ISE) method. Spike-recovery tests and certified reference materials (CRMs) will validate ICP-MS/ICP-OES trace metal detection limits in high-sulfate, low-pH topsoils.

Concurrently, clinical inter-rater reliability among examining dental professionals will be calibrated. Diagnostic consistency across Dean’s Index, the Developmental Defects of Enamel (DDE) index, and the DMFT index will be established via repeated independent examinations, requiring a minimum Cohen’s kappa coefficient of *κ* ≥ 0.80 prior to deployment. This Phase 0 framework provides a site-specific calibration mechanism that accounts for local geochemical variability and standardizes clinical diagnostic thresholds before scaling to the full cohort.

### 2.2. Study Area and Sample Size Determination

#### 2.2.1. Sample Size Justification and Power Calculation

Sample size determination was performed using G*Power (v3.1.9.7). Assuming a two-tailed multiple linear/logistic regression setup with up to 10 predictor variables, an alpha level of *α* = 0.05, and a target statistical power (1 − *β* = 0.80), the minimum required human cohort sample size was calculated as *N* = 330. Accounting for a predicted 15% non-response or drop-out rate, the final target clinical cohort size was fixed at *N* = 388 participants. The clinical cohort (*N* = 388) will be stratified into two sub-groups: a pediatric cohort (*n* = 194, aged 6–12 years) to directly evaluate active and recent amelogenesis disruption, and an adult cohort (*n* = 194, aged ≥18 years) to assess cumulative life-long dental caries burden and historical fluorosis retention.

The selected effect size (*f*^2^ = 0.15) was chosen as a conservative benchmark based on previous multi-contaminant environmental health studies in mining-impacted regions evaluating fluorosis severity and enamel hypoplasia against mixed heavy metal exposure. A sensitivity analysis conducted in G*Power demonstrates that with *N* = 388 participants, an *α* = 0.05, and 10 predictor variables, the study maintains >99% power to detect a medium effect size (*f*^2^ = 0.15) and 81.2% power to detect a small-to-medium effect size (*f*^2^ = 0.05), ensuring adequate statistical protection against Type II errors under subtle exposure-response relationships.

For the environmental matrix, *n* = 120 drinking water samples and *n* = 60 topsoil samples (collected in triplicate) will be sampled across eMalahleni, Barberton and Middelburg to ensure adequate spatial representation across study sectors. Water (*n* = 120) and topsoil (*n* = 60) sampling points will be distributed across a stratified grid network (2 km × 2 km cell density). This point density satisfies the minimum threshold required for Inverse Distance Weighting (IDW) spatial interpolation to minimize spatial prediction error.

To establish a comparative control baseline, an unexposed reference town, Mbombela (Nelspruit), situated in the same eco-geographical zone of Mpumalanga, but strictly outside active coal mining catchments, industrial effluent runoff, and Acid Mine Drainage (AMD) impact zones, has been incorporated into the sampling frame. Environmental sampling (*n* = 20 drinking water, *n* = 10 topsoil) and clinical screening (*N* = 60 residents) will be executed at this reference site using identical protocols to establish background elemental baselines and non-impacted dental morbidity rates.

#### 2.2.2. Proportional Spatial Sample Allocation

Sample distribution across the k selected study sites will follow a proportional population allocation strategy to prevent sampling bias toward lower-density rural or urban centers:

$$n_{i}=N\times \left(\frac{P_{i}}{P_{\mathrm{total}}}\right)$$

where *n_i_* represents the allocated sample size for town *i*, *N* is the total targeted sample size (*N* = 388 for residents; *n* = 120 for water samples; *n* = 60 for soil sites), *P_i_* is the population of town *i*, and *P*_total_ is the aggregate population across all selected study sites.

### 2.3. Field Sampling Protocol and Laboratory Preparation

#### 2.3.1. Drinking Water Sampling Protocol

Drinking water samples (*n* = 120) will be collected from primary domestic drinking water sources, including municipal taps, borehole hand-pumps, and storage containers across the designated sampling grids [12].

Samples will be collected in 500 mL acid-washed high-density polyethylene (HDPE) bottles [13]. Prior to collection, tap lines will be flushed for 2 to 3 min to clear standing water. Each container will be rinsed thrice with the source water before final collection. Water samples will be acidified on-site to pH < 2.0 using ultrapure nitric acid (HNO_3_, 65%, Suprapur, Merck, Darmstadt, Germany), preserved on ice during transit, and stored at 4 °C prior to digestion and laboratory elemental analysis [14,15].

#### 2.3.2. Topsoil Matrix Sampling Quantification

Topsoil samples (*n* = 60, collected in triplicate per sampling location) will be collected at a depth of 0–10 cm using a stainless-steel auger to characterize surface heavy metal accumulation and secondary exposure pathways [16]. Soil samples will be sealed in pre-labelled polyethylene bags, transported to the laboratory, air-dried at room temperature (25 ± 2 °C), sieved through a 2 mm stainless steel mesh, and stored in a desiccator prior to acid digestion [16].

### 2.4. Analytical Instrumentation and Quality Control

#### 2.4.1. Digestion and Trace Elemental Quantification

Water samples will undergo microwave-assisted acid digestion using ultrapure HNO_3_. Soil samples will be digested via open-vessel wet digestion using a 3:1 mixture of concentrated HCl and HNO_3_ (aqua regia).

Elemental concentrations including Fluoride (F^−^), Lead (Pb), Cadmium (Cd), Arsenic (As), Chromium (Cr), Iron (Fe), Manganese (Mn), Copper (Cu), and Zinc (Zn) will be quantified using Inductively Coupled Plasma Mass Spectrometry (ICP-MS, Agilent 7900) or Inductively Coupled Plasma Optical Emission Spectrometry (ICP-OES) [17]. Fluoride ion concentrations will be measured using an Ion-Selective Electrode (ISE, Thermo Scientific Orion) buffered 1:1 with TISAB IV (containing CDTA and tartrate to de-complex fluoride bound to ferric (Fe^3+^) and aluminum (Al^3+^) cations in AMD waters) [18,19].

#### 2.4.2. Quality Assurance and Quality Control (QA/QC)

- Rigorous QA/QC procedures will be enforced in accordance with ISO/IEC 17025 and IUPAC criteria:
- Reagent Blanks and Calibration Operational blanks and multi-element standard solutions will be analyzed every 10 samples to monitor instrument drift. Five-point linear calibration curves will maintain *R*^2^ ≥ 0.999.
- Limit of Detection (LOD) and Limit of Quantification (LOQ): LOD and LOQ will be established as 3.3 × (*σ*/*S*) and 10 × (*σ*/*S*), respectively, where *σ* is the standard deviation of 10 reagent blank responses and *S* is the slope of the calibration curve.
- Certified Reference Materials (CRMs): Analytical accuracy will be verified using NIST-certified water (SRM 1643f) and soil (SRM 2711a) reference materials, with target recovery rates required between 90% and 110%.
- Precision: Analytical precision will be confirmed through triplicate sample processing, maintaining a relative standard deviation (RSD) below 5%.
- Left-Censored Data Handling: Analytical results falling below the Limit of Detection (<LOD) will be imputed using maximum likelihood estimation (MLE) for regression modelling or substituted with $\mathrm{LOD}/\sqrt{2}$ for descriptive summary statistics when non-detects constitute <15% of the total sample set.

### 2.5. Environmental Exposure and Risk Assessment for Dental Endpoints

The chronic exposure and non-carcinogenic health risks associated with trace metal and fluoride exposure through drinking water, soil ingestion, and dust inhalation will be quantified using standard United States Environmental Protection Agency (US EPA) risk assessment methodologies [20]. Because the primary clinical endpoints centre on dental pathologies (dental fluorosis, developmental defects of enamel, and caries burden) rather than oncological outcomes, risk metrics focus strictly on threshold-based toxicity (*HQ* ≥ 1), chronic daily exposure levels, and exposure-response relationships [21].

#### 2.5.1. Chronic Daily Intake (*CDI*)

The Chronic Daily Intake via drinking water ingestion (*CDI*_ing_, mg/kg·day) for each target element is calculated via Equation (1) [22]:

(1) $${\mathrm{CDI}}_{\mathrm{Ing}}=\frac{C_{\mathrm{water}}\times IR_{\mathrm{water}}\times \mathrm{EF}\times \mathrm{ED}}{\mathrm{BW}\times \mathrm{AT}}$$

where *C*_water_ is the concentration of the trace element/fluoride in drinking water (mg/L); *IR*_water_ is the daily water ingestion rate (2.0 L/day for adults, 1.0 L/day for children); *EF* is exposure frequency (365 days/year); *ED* is exposure duration (70 years for adults, 6 years for children); *BW* is body weight (70 kg for adults, 15 kg for children); and *AT* is averaging time (*ED* × 365 days) [23].

To account for terrestrial exposure pathways highlighted in Section 3.1, Chronic Daily Intake via incidental soil ingestion (CDI_soil_, mg/kg·day) and dust inhalation (CDI_inh_, mg/kg·day) are quantitatively modeled using Equations (2) and (3):

(2) $${\mathrm{CDI}}_{\mathrm{soil}}=\frac{C_{\mathrm{soil}}\times IR_{\mathrm{soil}}\times \mathrm{EF}\times \mathrm{ED}}{\mathrm{BW}\times \mathrm{AT}}\times 10^{-6}$$

(3) $${\mathrm{CDI}}_{\mathrm{inh}}=\frac{C_{\mathrm{soil}}\times \mathrm{InhR}\times \mathrm{EF}\times \mathrm{ED}}{\mathrm{PEF}\times \mathrm{BW}\times \mathrm{AT}}$$

where *C*_soil_ represents topsoil concentration (mg/kg); *IR*_soil_ is the daily soil ingestion rate (100 mg/day for adults, 200 mg/day for children); *InhR* is the inhalation rate (20 m^3^/day for adults, 7.5 m^3^/day for children); and *PEF* is the particle emission factor (1.36 × 10^9^ m^3^/kg).

#### 2.5.2. Non-Carcinogenic Hazard Characterization

Systemic non-carcinogenic risk for individual trace elements is expressed as the Hazard Quotient (*HQ*), determined by Equation (4) [24]:

(4) $$HQ_{i}=\frac{{\mathrm{CDI}}_{i}}{\mathrm{Rf}D_{i}}$$

where RfD*_i_* is the oral reference dose (mg/kg·day) established by the US EPA Integrated Risk Information System (IRIS) or WHO guidelines. The Oral Reference Dose (RfD) for fluoride is set at 0.06 mg/kg·day, derived from US EPA IRIS database based on NOAEL thresholds for dental fluorosis.

To evaluate the cumulative toxicological pressure exerted by multi-element and multi-route exposure, the cumulative Hazard Index (HI) is calculated via Equation (5) [24]:

HI_total_ = HQ_water_ + HQ_soil_ + HQ_inhalation_ (5)

An HQ or HI < 1 indicates that total exposure remains below toxicological thresholds with minimal risk of adverse tissue effects. An HQ or HI ≥ 1 signifies that environmental exposure exceeds safe thresholds, serving as an indicator for heightened risk of trace-metal-induced toxicities and developmental enamel disruption.

### 2.6. Clinical Oral Health Examinations

Clinical examinations of the resident cohort (*N* = 388) will be conducted by calibrated dental clinicians under standardized natural/portable lighting conditions using disposable mouth mirrors and standard WHO ball-ended periodontal probes (0.5 mm ball tip), adhering strictly to WHO oral health survey methodologies [25]. Examining clinicians will be fully blinded to participants’ geographic origin, water source types, and environmental metal exposure levels during screening. A standardized intraoral photographic record (labial surfaces of maxillary and mandibular anterior teeth) will be captured for a random 10% subset of participants using a ring-flash-equipped intraoral camera for post-hoc diagnostic validation [26].

#### 2.6.1. Clinical Dental Indices

- Dental Fluorosis: Severity and prevalence of fluorosis will be classified using Dean’s Fluorosis Index (categorized as Normal, Questionable, Very Mild, Mild, Moderate, or Severe).
- Developmental Defects of Enamel: Enamel opacities and hypoplasia will be recorded using the Developmental Defects of Enamel (DDE) Index to distinguish non-fluoride opacity patterns from classical fluorosis
- Caries Burden: Dental caries experience will be quantified using the Decayed, Missing, and Filled Teeth (DMFT) index for permanent dentition (or dmft for primary dentition).

#### 2.6.2. Intra- and Inter-Examiner Calibration

Prior to field surveys, all participating dental clinicians will undergo a two-day calibration training program led by a senior pediatric dentist using representative clinical photographic benchmarks. Intra-examiner and inter-examiner reliability will be evaluated on a calibration subset of 20 non-study individuals examined twice, 7 days apart [27]. Inter- and intra-examiner agreement for Dean’s Index, DDE Index, and DMFT status will be quantified using Cohen’s kappa (*κ*) coefficient [28]. A threshold of *κ* ≥ 0.80 will be required prior to field deployment to ensure high diagnostic consistency and minimize classification bias [29].

### 2.7. Statistical Modeling and Confounder Control

#### 2.7.1. Descriptive and Spatial Analysis

Data normality will be verified using Kolmogorov-Smirnov and Shapiro-Wilk tests [30]. Continuous variables (water element concentrations, *CDI*, DMFT) will be expressed as mean ± standard deviation (SD) or median (interquartile range [IQR]). Categorical clinical indices (Dean’s Index classifications) will be summarized as frequencies and percentages [31]. Spatial variation across study locations will be evaluated using One-Way Analysis of Variance (ANOVA) or Kruskal-Wallis tests. Geospatial interpolation and contamination mapping using Inverse Distance Weighting (IDW) will be executed using ArcGIS Pro (v3.1, Esri Inc., Redlands, CA, USA) or QGIS (v3.28 LTR).

#### 2.7.2. Regression Modeling Against Clinical Outcomes

Statistical modelling will match the explicit distribution of each primary clinical outcome variable (*Y*):

- Caries Burden (DMFT/dmft Count Data): Modeled using Poisson regression or Negative Binomial regression (or Zero-Inflated Negative Binomial regression in the event of excess zeros) to account for over-dispersion.
- Dental Fluorosis Severity (Dean’s Index—Ordinal): Modeled using Ordinal Logistic Regression (Proportional Odds Model).
- Enamel Hypoplasia & Opacities (DDE Index—Binary/Multinomial): Modeled using Binary or Multinomial Logistic Regression.

Predictor streams (*X*) will evaluate direct exposure concentrations (*C*_water_, *C*_soil_), Chronic Daily Intakes (CDI_Ing_), Hazard Quotients (HQ), and cumulative Hazard Indices (HI_total_).

#### 2.7.3. Confounder Control and Covariate Adjustment

To isolate the independent effects of drinking water contaminants on dental pathologies, all regression models will adjust for key biological, socioeconomic, and dietary covariates:

- Demographics & Residency: Age, gender, and length of continuous residence.
- Socioeconomic Status (SES): Composite wealth index derived from household income, education level, and employment.
- Oral Hygiene Habits: Daily tooth brushing frequency, fluoride toothpaste utilization, and access to routine dental care
- Nutritional & Dietary Factors: Daily dietary calcium and vitamin D intake evaluated using a validated semi-quantitative Food Frequency Questionnaire (FFQ).

Statistical significance is set at *α* = 0.05 (two-tailed). All statistical operations will be executed using IBM SPSS Statistics (v28.0).

## 3. Discussion

The integration of multi-matrix environmental geochemistry with clinical oral epidemiology established in this protocol addresses a critical systemic vulnerability in South African public health infrastructure [32]. Historically, environmental health assessments in mining regions have operated within siloed frameworks, focusing either strictly on ecological geochemistry or isolated clinical surveys without mapping spatial exposure dynamics. This protocol provides a structured methodology to operationalize the Source-Pathway-Receptor-Impact (SPRI) model, bridging the gap between geochemical contamination profiles and structural tooth pathologies in vulnerable, mining-adjacent populations.

### 3.1. Environmental Exposure Pathways and Source Variability

Environmental exposure to heavy metals and halogens in mining-impacted regions such as Mpumalanga is governed by the interaction between natural geochemical conditions and anthropogenic mining activities [33,34]. Acid mine drainage (AMD), a well-documented consequence of coal and gold mining, promotes the mobilization of sulphate-rich, low-pH effluents that enhance the solubility and transport of trace metals including lead (*Pb*), cadmium (*Cd*), chromium (*Cr*), manganese (*Mn*), and iron (*Fe*) [35,36].

Although centralized municipal treatment facilities are generally effective in reducing particulate loads and ensuring compliance with national drinking water standards, secondary contamination may occur within distribution networks after treatment [37]. Aging water reticulation infrastructure is particularly susceptible to internal corrosion processes, which facilitate the leaching of metal ions into drinking water at the point of consumption [37,38]. These processes are exacerbated by stagnation, hydraulic pressure fluctuations, and prolonged residence time within distribution systems, all of which increase the dissolution of metals from pipe materials into potable water [38].

In rural and peri-urban settings, reliance on shallow and unregulated groundwater sources further increases exposure risk. These boreholes are highly vulnerable to AMD infiltration and diffuse geogenic contamination [39,40]. The acidic, sulphate-rich nature of AMD reduces groundwater pH and enhances the mobility and bioavailability of toxic metals through increased mineral dissolution and reduced adsorption capacity in surrounding soils and aquifers [40].

Residential topsoil represents an additional and often under-characterized exposure pathway. In mining-impacted environments, soils act as long-term sinks for industrial emissions and tailings-derived particulates [41]. During dry seasonal periods, these particles are readily resuspended through wind erosion and anthropogenic disturbance such as heavy truck traffic along mining routes [42]. Resultant airborne particulate matter may be inhaled or ingested, particularly among children, contributing to cumulative multi-route exposure to toxic elements [43].

### 3.2. Biological Mechanisms Altering Amelogenesis: The Cocktail Effect

Amelogenesis is a tightly regulated biological process responsible for enamel formation and mineralization during tooth development. This process depends on precise calcium ion (*Ca*^2+^) transport and controlled enzymatic degradation of enamel matrix proteins, particularly amelogenin, mediated by matrix metalloproteinase-20 (MMP20) and kallikrein-4 (KLK4) [44].

**Figure 1:**
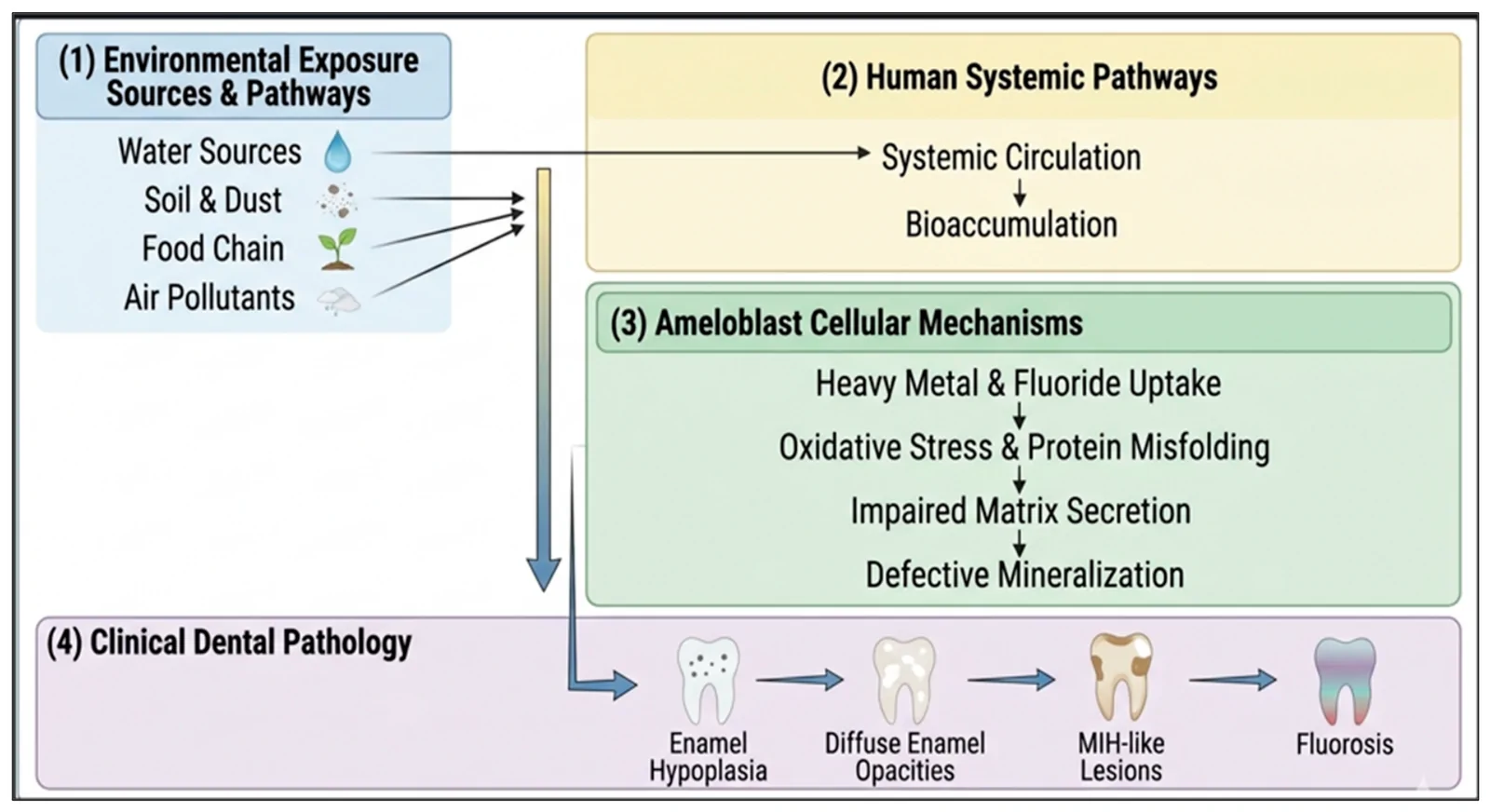
Hypothesized conceptual framework illustrating the multi-matrix environmental exposure pathways of heavy metals and fluorides, their proposed biological mechanisms within secretory ameloblasts, and hypothesized clinical dental pathology presentations.

Concurrent exposure to fluoride and heavy metals has been proposed to produce synergistic toxicological interactions that disrupt enamel biomineralization. Excess fluoride exposure during enamel maturation interferes with amelogenin degradation, resulting in subsurface hypomineralization, increased porosity, and clinically visible enamel opacities consistent with dental fluorosis [45,46]. These manifestations are commonly classified using Dean’s Fluorosis Index. To visually map how these complex environmental routes translate into intracellular stress, a comprehensive overview of the target mechanisms is provided in Figure 1.

Heavy metals such as Pb^2+^ and Cd^2+^ may further disrupt enamel formation by substituting for Ca^2+^ within hydroxyapatite crystal lattices due to similarities in ionic radius and charge [47]. This substitution impairs crystal growth, weakens enamel prism structure, and increases susceptibility to acid-mediated demineralization.

At the cellular level, heavy metal exposure has been associated with oxidative stress and disruption of intracellular organelle function, including endoplasmic reticulum stress in mineralizing cells [48]. Such effects may impair ameloblast function, thereby altering enamel matrix secretion and maturation.

The combined exposure to fluoride and heavy metals may therefore represent a multi-factorial disruption pathway affecting enamel development. Epidemiological evidence supports associations between environmental exposure to such contaminants and increased prevalence of dental caries (DMFT index), enamel defects (Developmental Defects of Enamel, DDE), and fluorosis in affected populations [49].

### 3.3. Comparative Analysis of Mining-Impacted Regions

To contextualize the methodological framework against broader international findings, Table 1 provides a comparative summary of international environmental case studies linking mining-induced geochemical contamination with clinical oral pathologies. The scientific value of this methodology is further reinforced when contextualized against structural findings from international peer-reviewed case studies. For instance, recent work in endemic fluorosis zones of southeastern Türkiye explicitly demonstrated the coupling of US EPA Hazard Quotients (*HQ*) with clinical fluorosis prevalence across pediatric cohorts, providing a direct methodological precedent for our integrated HRA–oral health framework. These comparisons demonstrate the global relevance of integrating geochemical data with clinical oral indexing in heavily industrialized zones:

By aligning with these global frameworks, this protocol expands on their findings by integrating multi-matrix environmental sampling (water and soil) directly with multi-index clinical dental screening within a single, unified predictive regression structure.

### 3.4. Policy Integration and Methodological Limitations

From a public health perspective, this framework aligns with the United States Environmental Protection Agency (US EPA) Health Risk Assessment (HRA) model, which converts environmental concentration data into standardized risk metrics such as the Hazard Index (HI) [49]. An HI value greater than 1 is commonly interpreted as indicative of potential non-carcinogenic health risk, providing a quantitative basis for regulatory decision-making.

In the South African regulatory context, findings may inform compliance and intervention strategies aligned with the South African National Standard (SANS 241) for drinking water quality [49]. This includes potential infrastructure upgrades, improved point-of-use treatment systems, and targeted oral health surveillance in high-risk communities.

However, methodological limitations must be acknowledged. The cross-sectional design restricts causal inference, as exposure and outcome data are captured at a single time point. This is particularly relevant for amelogenesis, which occurs primarily during early childhood; thus, current environmental measurements may not accurately reflect historical exposure conditions. In addition, dietary intake, nutritional status (calcium and vitamin D levels), and oral hygiene practices may act as confounding variables influencing clinical outcomes independently of environmental exposure.

To mitigate these limitations, the study employs multivariate regression modelling and spatial interpolation techniques such as inverse distance weighting (IDW) to improve exposure estimation and control for confounding factors [50]. While these approaches improve analytical robustness, residual uncertainty in historical exposure reconstruction remains unavoidable.

## 4. Conclusions

This study protocol establishes a comprehensive, multi-matrix framework designed to bridge the historical gap between environmental geochemistry and clinical oral epidemiology in mining-impacted regions. By operating under the Source-Pathway-Receptor-Impact (SPRI) model, the proposed methodology moves beyond single-variable monitoring to simultaneously evaluate exposure dynamics across drinking water networks and terrestrial top-soil sinks. This integrated structure allows researchers to model the cumulative non-carcinogenic health hazards and systemic “cocktail effects” that heavy metal mixtures and halogens exert on human amelogenesis.

The systematic deployment of this protocol in the coal and gold mining sectors of Mpumalanga, South Africa, will generate high-resolution geospatial contamination maps and robust, confounding-controlled multivariate regression models. These empirical outputs will isolate the true environmental determinants of localized dental fluorosis, developmental defects of enamel (DDE), and dental caries experiences. Ultimately, this research provides a scalable, legally defensible scientific baseline capable of guiding local water boards, environmental health practitioners, and municipal planners. The resulting datasets will support targeted public health interventions and justify crucial water quality policy adjustments to protect vulnerable communities situated adjacent to heavy industrial operations globally.

## 5. Reagents Setup

To ensure strict analytical reproducibility, data validation, and cross-laboratory standardization, all technical reagents, calibration standards, and clinical tools must be prepared and maintained according to the precise specifications detailed below.

### 5.1. Analytical Chemistry Reagents and Calibration Standard Preparation

- Water Purification Infrastructure: All laboratory-grade water used for reagent dilution, vessel cleaning, and blank preparation shall consist of ultrapure water produced by a Milli-Q purification system (Millipore, Bedford, MA, USA), with a resistivity of 18.2 MΩ·cm at 25 °C.
- Acid Digestion Matrices: Analytical-grade nitric acid (HNO_3_, 65% *v*/*v*, Suprapur, Merck, Darmstadt, Germany) shall be used for field sample preservation and microwave-assisted soil digestion procedures. A 2% (*v*/*v*) HNO_3_ rinsing solution shall be prepared daily by diluting 20 mL of Suprapur HNO_3_ with 980 mL of Milli-Q water. This solution shall serve as both the ICP-MS carrier matrix and sample rinse solution.
- Multi-Element Calibration Standards: High-purity, NIST-traceable multi-element stock calibration standards (100 mg/L containing Lead (*Pb*), Cadmium (*Cd*), Chromium (*Cr*), Manganese (*Mn*), and Arsenic (*As*); Devenish Solutions, South Africa) shall be stored at 4 °C. Working calibration standards shall be prepared by serial dilution in 2% HNO_3_ to generate a five-point linear calibration curve spanning 0.1 µg/L to 100 µg/L.
- Internal Calibration Standards: An internal standard solution containing Rhodium (Rh), Indium (In), and Bismuth (Bi) at a working concentration of 10 µg/L shall be continuously introduced through a secondary peristaltic pump line to monitor and correct for instrumental drift and matrix suppression during ICP-MS operation.

### 5.2. Halogen Analysis Buffer Setup (Fluoride ISE)

- **Total Ionic Strength Adjustment Buffer (TISAB II):** To minimize interference from complexing divalent iron (Fe^2+^), ferric (Fe^3+^), and aluminum (Al^3+^) ions while stabilizing ionic strength, TISAB IV (containing CDTA and tartrate) shall be prepared by dissolving 58 g Sodium Chloride (NaCl), 57 mL glacial acetic acid (CH_3_COOH), and 4 g Cyclohexylenedinitrilotetraacetic Acid (CDTA) in 500 mL Milli-Q water. The pH shall be adjusted to 5.3–5.5 using 5 M Sodium Hydroxide (NaOH), after which the solution shall be diluted to a final volume of 1.0 L in a volumetric flask.
- **Fluoride Stock Solutions:** A primary fluoride stock solution (100 mg/L F^−^) shall be prepared by dissolving exactly 0.2210 g of pre-dried analytical-grade Sodium Fluoride (NaF) in Milli-Q water and diluting to 1000 mL. Working calibration standards of 0.1, 0.5, 1.0, 5.0, and 10.0 mg/L shall subsequently be prepared and mixed 1:1 with TISAB IV prior to fluoride ion-selective electrode (ISE) calibration.

### 5.3. Decontamination of Collection Vessels

- All reusable high-density polyethylene (HDPE) sampling bottles, borosilicate glassware, and plastic soil scoops shall undergo a standardized decontamination protocol consisting of a 24-h immersion in 10% (*v*/*v*) analytical-grade HNO_3_, followed by three sequential rinses with Milli-Q water. All equipment shall then be air-dried inside a certified Class 100 laminar-flow clean bench prior to field deployment.

### 5.4. Clinical Survey Materials and Equipment Setup

- **Portable Clinical Examination Kits:** Field dental examination kits shall include sterilized diagnostic packs containing a front-surface dental mirror, a single-use WHO periodontal probe with a 0.5 mm ball tip and black reference bands at 3.5 mm and 5.5 mm [8], and sterile cotton rolls.
- **Artificial Field Lighting System:** Portable intraoral LED headlamps providing a color temperature range of 4500–5500 K and illumination intensity ≥ 25,000 lux shall be used during all clinical examinations to ensure standardized visualization conditions and minimize ambient lighting variability during field assessments.

## Figures and Tables

**Table 1:** Comparative summary of international environmental case studies linking mining-induced geochemical contamination with clinical oral pathologies.

Geographic Case Study	Identified Environmental Stressors	Human Clinical Oral Exam Performed?	Documented Clinical Oral Pathologies	Methodological Alignment with Current Protocol
Mpumalanga Mining Districts, South Africa	Elevated *F*^−^, *Pb*, *Cd*, *Cr*, *Mn*, and *As* across drinking water and topsoil matrices.	Yes (Planned)	Dental fluorosis, structural DDE changes, and heightened caries experiences (DMFT).	High-resolution spatial profiling (IDW) coupled with Ordinal Logistic and Multiple Linear Regression.
Southeastern Türkiye Fluorosis Zone	High *F*^−^ in drinking water sources across rural groundwater catchments.	Yes	Clinical dental fluorosis was assessed via Dean’s Index across pediatric cohorts.	Direct alignment: Coupling of US EPA *CDI* and *HQ* metrics with clinical fluorosis prevalence across age groups.
Asturias Mercury-Mining District, Spain	High soil and water contamination of Hg, As, and Pb from historical mining tailings	No	Chronic systemic absorption; enamel coloration and increased friability in localized resident cohorts.	Focus on heavy metal transport from tailing deposits into domestic catchment basins via ecological mapping.
Panasqueira Wolfram Mine Region, Portugal	Severe *As*, *Cd*, *Cu*, and *W* concentrations in local soils and river systems	No	Increased bioaccumulation in local trophic chains; soft tissue alterations and structural hard tissue loss.	Utilization of advanced geostatistical spatial analysis to delineate primary human exposure zones adjacent to processing plants.
Livingstonia Coalfield Region, Malawi	Acid Mine Drainage (AMD) carrying excessive concentrations of *Fe*, *Mn*, and *SO*_4_^2−^ into local surface waters.	No	Highly corrosive point-of-use tap water; aesthetic consumer rejection and accelerated systemic dental wear.	Geochemical characterization of coal drainage impacts on local domestic water networks.

## Data Availability

No new data were created or analyzed in this study. Data sharing is not applicable to this article.

## Abbreviations

The following abbreviations are used in this manuscript:

AMD	Acid Mine Drainage
CDI	Chronic Daily Intake
DDE	Developmental Defects of Enamel
DMFT	Decayed, Missing, and Filled Teeth (Permanent Dentition)
dmft	decayed, missing, and filled teeth (Primary Dentition)
FFQ	Food Frequency Questionnaire
HRA	Human Health Risk Assessment
ICP-MS	Inductively Coupled Plasma Mass Spectrometry
ICP-OES	Inductively Coupled Plasma Optical Emission Spectrometry
IDW	Inverse Distance Weighting
IQR	Interquartile Range
ISE	Ion-Selective Electrode
LOD	Limit of Detection
LOQ	Limit of Quantification
RfD	Oral Reference Dose
SANS	South African National Standards
SD	Standard Deviation
THQ	Target Hazard Quotient
TISAB	Total Ionic Strength Adjustment Buffer
